# Effect of Alpha-1 Antitrypsin on CFTR Levels in Primary Human Airway Epithelial Cells Grown at the Air-Liquid-Interface

**DOI:** 10.3390/molecules26092639

**Published:** 2021-04-30

**Authors:** Frauke Stanke, Sabina Janciauskiene, Stephanie Tamm, Sabine Wrenger, Ellen Luise Raddatz, Danny Jonigk, Peter Braubach

**Affiliations:** 1 Department of Pediatric Pneumology, Neonatology and Allergology, Hannover Medical School, Carl-Neuberg-Str. 1, 30625 Hannover, Germany; Tamm.Stephanie@mh-hannover.de (S.T.); Ellen.L.Raddatz@stud.mh-hannover.de (E.L.R.); 2 Biomedical Research in Endstage and Obstructive Lung Disease Hannover (BREATH), German Centre for Lung Research, Carl-Neuberg-Str. 1, 30625 Hannover, Germany; Janciauskiene.Sabina@mh-hannover.de (S.J.); Wrenger.Sabine@mh-hannover.de (S.W.); Jonigk.Danny@mh-hannover.de (D.J.); Braubach.Peter@mh-hannover.de (P.B.); 3 Department of Respiratory Medicine, Hannover Medical School, Carl-Neuberg-Str. 1, 30625 Hannover, Germany; 4 Institute of Pathology, Hannover Medical School, Carl-Neuberg-Str. 1, 30625 Hannover, Germany

**Keywords:** alpha1-antitrypsin, CFTR, epithelial cells, cystic fibrosis, air-liquid-interface culture

## Abstract

The cystic fibrosis transmembrane conductance regulator (CFTR) gene is influenced by the fundamental cellular processes like epithelial differentiation/polarization, regeneration and epithelial–mesenchymal transition. Defects in CFTR protein levels and/or function lead to decreased airway surface liquid layer facilitating microbial colonization and inflammation. The *SERPINA1* gene, encoding alpha1-antitrypsin (AAT) protein, is one of the genes implicated in CF, however it remains unknown whether AAT has any influence on CFTR levels. In this study we assessed CFTR protein levels in primary human lung epithelial cells grown at the air-liquid-interface (ALI) alone or pre-incubated with AAT by Western blots and immunohistochemistry. Histological analysis of ALI inserts revealed CFTR- and AAT-positive cells but no AAT-CFTR co-localization. When 0.5 mg/mL of AAT was added to apical or basolateral compartments of pro-inflammatory activated ALI cultures, CFTR levels increased relative to activated ALIs. This finding suggests that AAT is CFTR-modulating protein, albeit its effects may depend on the concentration and the route of administration. Human lung epithelial ALI cultures provide a useful tool for studies in detail how AAT or other pharmaceuticals affect the levels and activity of CFTR.

## 1. Introduction

Cystic fibrosis transmembrane conductance regulator (CFTR) is an epithelial anion channel critical for chloride and bicarbonate transport in many organs, including the lungs, liver, intestine and pancreas. CFTR is a multi-domain membrane protein, a member of adenine nucleotide-binding cassette transporters consisting of two transmembrane domains, two nucleotide binding domains and a unique regulatory domain [1]. It is expressed in the airway surface epithelium, submucosal glands and other epithelial organs [2]. Defective levels and function of CFTR leads to the impaired mucociliary clearance, loss of airway antimicrobial activity, persistent infection and progressive lung disease [3]. Cystic fibrosis (CF) caused by mutations in the *CFTR* gene remains one of the most fatal hereditary disorders worldwide. The pathologic features of CF include abnormal mucus production, chronic infections and neutrophil-dominant airway inflammation [4].

Current therapeutic approaches focus on restoring airway hydration and correcting defective levels of CFTR protein [3]. Some studies provide evidence that modifier genes associated with epithelial damage and repair can affect levels of CFTR protein and/or gene expression [5]. Among others, *SERPINA1* gene encoding AAT protein has been identified as one of the CF modifiers [6], and AAT has been characterized as an interaction partner of CFTR [7].

Human AAT is an acute phase glycoprotein (normal plasma concentration about 1–2 g/L), one of the best inhibitors of neutrophil elastase and proteinase 3 but also expresses broad immunomodulatory effects [8]. It is well recognized that individuals with severe inherited AAT deficiencies possess an increased risk for developing early onset lung emphysema [9]. The deficiency of AAT may also increase the risk for CF [6] albeit not all studies have confirmed this association [10]. 

Since decades human plasma purified preparations of AAT are used as a specific intravenous therapy for emphysema patients with AAT deficiency [11]. The efficacy of different doses of inhaled AAT has also been tested in CF patients [12,13,14]. Although AAT therapy is safe and significantly reduces elastase activity, there is still a lack of definite clinical evidence whether inhaled or intravenous AAT supplementation has beneficial effects in CF. Moreover, the impact of AAT protein on the CFTR levels has not been investigated. If it could be confirmed that exogenous AAT affects CFTR protein levels and/or functional activity, one could predict that the therapy with AAT benefits patients with defective CFTR. It is difficult to conduct appropriate clinical study in terms of patient’s selection with rare disease, choosing relevant dosage and duration of AAT treatment and endpoints assessments. Therefore, in this study we employed primary human lung epithelial cells grown at the air-liquid-interface (ALI) to study AAT effects on CFTR levels in vitro.

## 2. Results

### 2.1. Characterization of ALI Cultures

**Figure 1:**
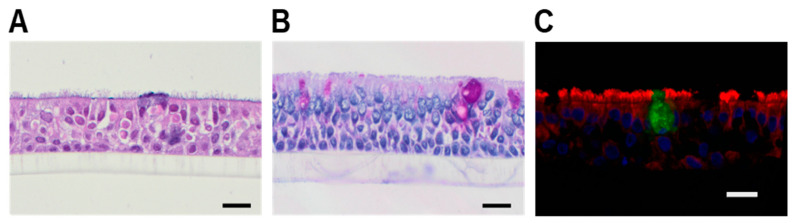
Histological examination reveals that bronchial epithelial cells cultured at ALI differentiated and form a pseudostratified epithelium with cialiated and mucus-producing cells at the apical side. Cells obtained from human trachea (donor lung) were expanded for 6 days according to the protocol of The University of Leiden [15] and afterwards differentiated at ALI for 22 days using Pneumacult ALI medium until cilia beating was observed. (**A**) Formalin fixed and paraffin embedded primary bronchial epithelial cell ALI inserts were stained with hematoxylin and eosin (H&E). (**B**) Methacarn fixed and paraffin embedded primary bronchial epithelial cell ALI inserts were stained with periodic acid-Schiff (PAS) and hematoxylin. (**C**) Polarized distribution of ciliated cells stained for acetylated tubulin (red) and presence of a MUC5AC positive goblet cell (green) in ALI cultures of differentiated bronchial epithelial cells. Formalin fixed and paraffin embedded primary bronchial epithelial cell ALI inserts were stained with specific antibodies for acetylated tubulin and MUC5AC. 4′,6-diamidino-2-phenylindole (DAPI, blue) was used for nuclear staining. Scale bars represent 20 µm.

The crucial steps determining the success of growing primary human cells into fully differentiated pseudostratified epithelium depend on the expansion and differentiation media, which contain specific reagents. In this study, cell isolation and propagation we performed based on the very well-established protocols from The University of Leiden, The Netherlands [15] and from The University of North Carolina at Chapel Hill [16]. The ALI epithelia looked comparable using both protocols. Pseudostratified, fully differentiated epithelial layers were formed within 4 weeks (Figure 1). The presence of goblet cells was confirmed by using periodic acid-Schiff (PAS) staining (Figure 1B) and by the identification of MUC5AC positive cells (Figure 1C). Ciliated cells were identified from the staining of acetylated tubulin (red) on the apical surface (Figure 1C).

### 2.2. Methodological Approaches for Analysis of ALI Cultures

**Figure 2:**
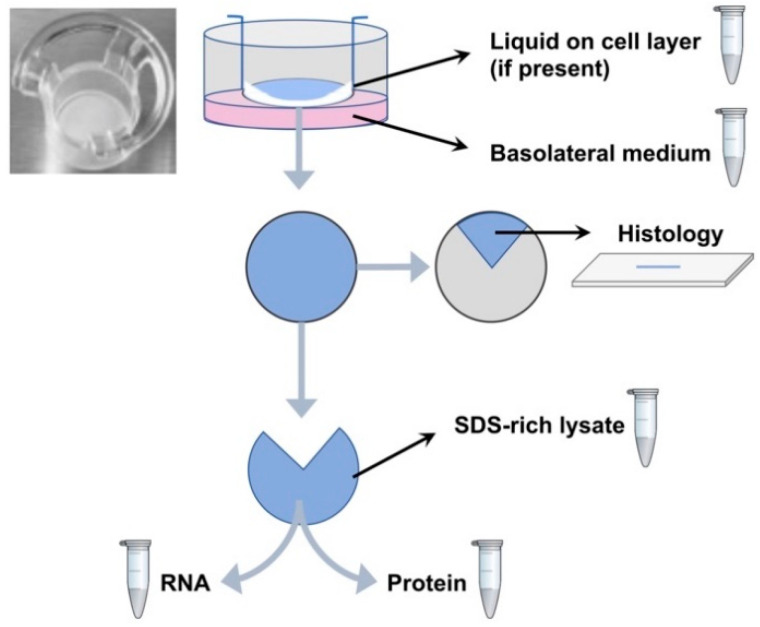
The analysis of bronchial epithelial cell ALI cultures. Six inserts were generated from one donor trachea ring of approximately 5 mm width. The samples from a single insert were collected for protein and histological analyses and for gene expression. The data obtained for the same insert utilizing different methodologies allow better characterization of patient’s epithelial cells and the comparison between patients. Sampling of material for protein analysis was performed separately for the outermost rim of the insert and for the remaining central part (see methods for details).

Our next goal was to show how one can optimize ALI’s use. As illustrated in Figure 2, from one insert we were able to generate material for carrying multiple analyses. We processed every insert as follows:

- Collected apical supernatants and basolateral medium;
- Scraped with a rhino-pro curette cells from the outermost rim of the insert (where mucous material has accumulated) and stored in lysis buffer for protein analysis;
- Approximately 3/4 of the insert was scraped with a curette for the RNA isolation (recovery of 300 to 600 pg/µL RNA, with integrity values of 7.1 to 7.7) and for the whole-cell lysates preparation in order to obtain the solubility of the highly hydrophobic CFTR protein. Protein yield varied considerably among lysates (from 1 to 12 mg/mL) but was always sufficient for at least one analysis;
- Approximately 1/4 of the remaining insert was fixed in formalin or methacarn and paraffin embedded for the immunohistochemistry analysis.

### 2.3. Immunostainings of CFTR in ALI Cultures in Comparison to Human Bronchus Tissue

**Figure 3:**
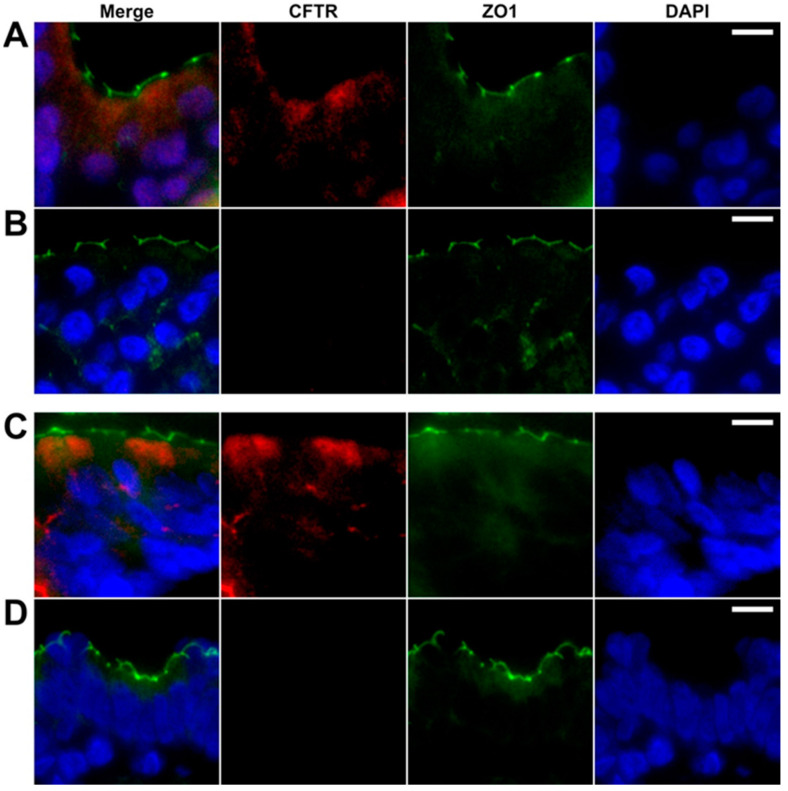
Staining of ALI inserts and human bronchus tissue for CFTR. Formalin fixed and paraffin embedded, primary bronchial epithelial cell ALI inserts (**A**,**B**) and human bronchus tissues (**C**,**D**) were stained with specific antibodies for CFTR (red) and ZO1 (green), and with DAPI (blue). (**A**) ALI inserts and (**C**) human bronchus epithelia showed cytoplasmic and membranous staining for CFTR with predominance in the apical compartment along with typical apico-lateral ZO1 positivity. Pre-incubation of the primary antibody with a CFTR specific peptide completely blocked the CFTR signal (**B**,**D**) whereas no apparent change occurred in ZO1 reactivity. Scale bars are 10 µm, respectively.

One of the crucial aspects when evaluating the airway models is the presence of the CFTR. Therefore, formalin fixed and paraffin embedded fully differentiated bronchial epithelial ALI cultures and human bronchus tissues were stained in parallel with specific antibodies for CFTR, zonula occludes protein 1 (ZO1) and with DAPI, as a nuclear counterstain. In both, ALI cultures and native human bronchial epithelium, we found the cytoplasmic and membranous staining for the CFTR, with a predominance in the apical compartment, along with a typical apico-lateral ZO1 positivity (Figure 3). The pre-incubation of primary antibody with a specific peptide of CFTR (at the concentration of 1 µg/µL) completely blocked the CFTR signal with no apparent change in ZO1 reactivity (Figure 3). Even when the peptide was diluted by three orders of the magnitude, the CFTR signal was absent (data not shown), confirming the specificity of the monoclonal anti-CFTR antibody.

### 2.4. Differential Staining of CFTR Protein in Human Bronchial Epithelial ALI Cultures

**Figure 4:**
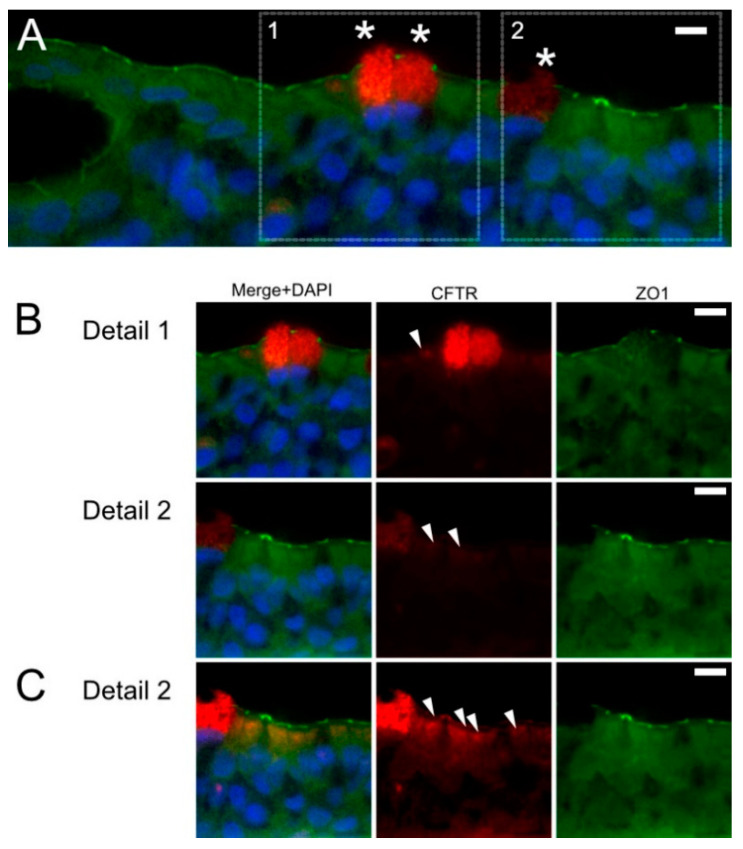
Differential CFTR staining in human epithelial ALI inserts. Sections of formalin fixed and paraffin embedded epithelial cell ALI cultures were double immuno-stained for CFTR (red) and ZO1 (green). Cell nuclei were visualized with DAPI (blue). (**A**) A small number of cells with a high CFTR content was detected (*). (**B**) When adjusting brightness and contrast to include these CFTR-high cells, the typical apical signal in the other epithelia was barely seen (arrowhead). (**C**) After linear adjustment of brightness and contrast, we were able to observe CFTR positive cells. Cell nuclei were visualized with DAPI (blue). Scale bars are 10 µm.

The sections of formalin fixed and paraffin embedded ALI cultures were double-stained for the CFTR and ZO1. In all investigated ALI cultures, we observed a small number of cells with high CFTR content (Figure 4A). Notably, when adjusting brightness and contrast to include the CFTR-high cells, the typical apical signal was barely seen (Figure 4B, arrowheads). After linear adjustment of brightness and contrast, we observed approximately 10 times weaker signal (Figure 4C).

**Figure 5:**
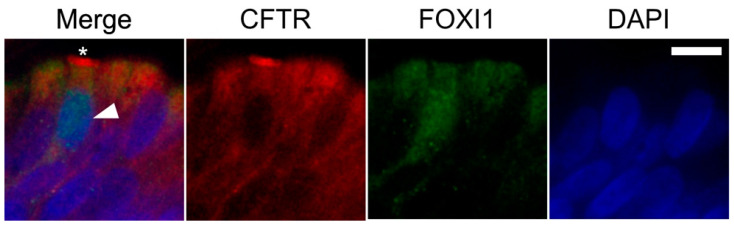
Human bronchial epithelial cell staining for FOXI1/CFTR. Sections of formalin fixed and paraffin embedded human bronchus were immunostained for CFTR (red) and FOXI1 (green). Cell nuclei were visualized with DAPI (blue). Rare cells with nuclear positivity for FOXI1 (arrowhead) and intense apical positivity for CFTR (*)–compatible with pulmonary ionocytes-could be observed. Scale bar is 10 µm.

When sections of formalin fixed and paraffin embedded human bronchus were immuno-stained for CFTR and Forkhead Box I1 (FOXI1), we found rare cells with nuclear positivity for FOXI1 and intense apical positivity for CFTR, compatible with pulmonary ionocytes (Figure 5). Taken together, we show that ALI cultures well represent human bronchial epithelium with regard to CFTR positive epithelial cells.

### 2.5. Effect of AAT on CFTR Levels in T84 Cells

**Figure 6:**
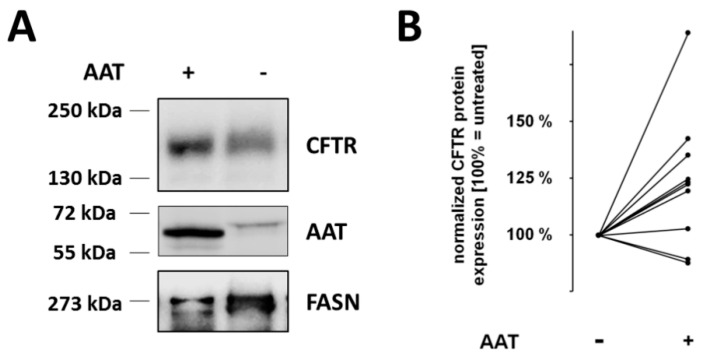
Increased CFTR expression in T84 cells treated with AAT. (**A**) T84 cells were incubated overnight alone or in the presence of AAT (0.5 mg/mL). SDS-rich whole cell lysates were prepared and separated on a denaturing 6% polyacrylamide gel at 4 °C for 18 h until the 72 kDa size marker band approached the end of the acrylamide matrix. Electrophoretically separated proteins were transferred to nitrocellulose membrane and the CFTR was detected by using a mixture from four monoclonal anti CFTR antibodies (1:1:1:1). As a loading control, we used FASN (Fatty Acid Synthase). We show a representative blot from one out of ten different experiments. (**B**) Densitometry analysis of Western blot data. Values were normalized to the control (no treatment with AAT) whereby control and AAT-treated samples were derived from the same experiment. As such, the control and AAT-treated samples technically represent pairs. The signal in the untreated controls was defined as 100% and changes upon AAT-treatment are reflected by the deviation from 100%, whereby in eight out of ten cases an increase of CFTR expression was observed (*p* = 0.02, *n* = 10, Wilcoxon signed rank test).

To determine any putative effect of AAT on the CFTR levels, we initially used human intestinal cancer epithelial cell line T84, highly expressing CFTR, and historically used in experiments to study CFTR levels [17]. We incubated T84 cells over night with AAT (0.5 mg/mL) or with phosphate buffered saline (PBS) for non-treated control. As illustrated in Figure 6A, analysis of whole-cell lysates by Western blots utilizing a cocktail of four anti-CFTR-specific antibodies revealed that CFTR levels in T84 cells treated with AAT are higher than in non-treated controls. As expected, lysates prepared from AAT-treated cells were also positive for AAT protein (Figure 6A). When levels of CFTR were judged by densitometry of paired AAT/PBS samples from repeated experiments, an increase in CFTR protein was observed by about 25%–35% (*p* = 0.02, *n* = 10, Wilcoxon signed rank test) in AAT-treated versus PBS control cells (Figure 6B). 

### 2.6. AAT Regulates CFTR Levels in Human Bronchial Epithelial ALI Cultures

**Figure 7:**
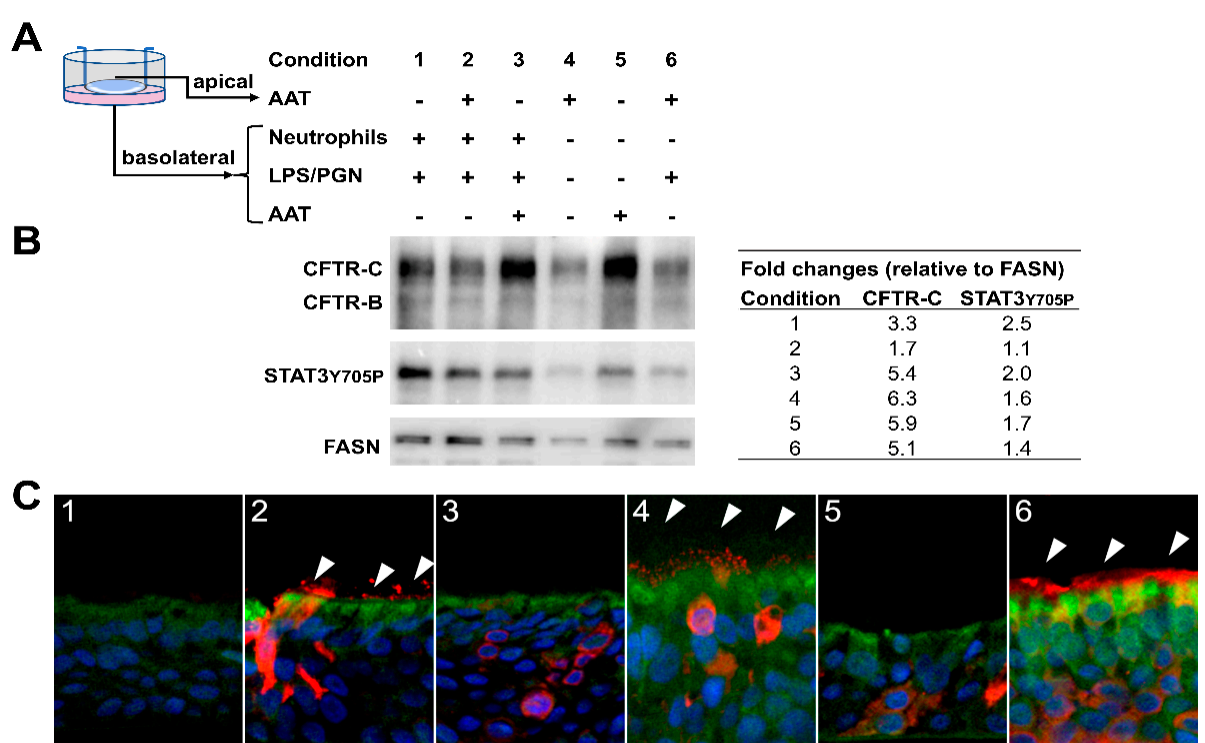
The effects of AAT on CFTR and STAT3 Y705P levels in ALI cultures generated from one donor trachea. The experiment was conducted after 19 days of ALI culture whereby cilia motion was visible after 12 days under the microscope. Representative data are shown out of six independent ALI inserts. (**A**) AAT alone and in combination with lipopolysaccharide (LPS) and peptidoglycan (PGN) or with neutrophils (2.5 × 10^6^ per well) was added on the top (apical) of ALI inserts or in the culture medium (basolateral). (**B**) Representative Western blot shows that basolateral AAT addition upregulates CFTR protein and slightly downregulates STAT3 Y705P levels. By contrast, apical AAT addition downregulates CFTR and STAT3Y705P levels. FASN (Fatty Acid Synthase) was used as a loading control. (**C**) Combined immunofluorescence stainings for CFTR (green) and AAT (red). Cell nuclei were visualized with DAPI (blue). AAT positive cells could be observed in cultures supplemented with AAT from the apical side (inserts 2, 4 and 6) and in cultures supplemented with AAT from the basolateral side (inserts 3 and 5) (red, marked by arrowheads). This was not the case in the ALI insert cultured without AAT (insert 1). Positivity for CFTR was observed in all ALI inserts (green).

Next, we assessed the ability of AAT to affect CFTR levels in primary bronchial epithelial ALI cultures at baseline or when cells were activated with pro-inflammatory stimuli. The AAT concentration was selected based on the assumption that in the epithelial lining fluid of the lower respiratory tract AAT constitutes about 10% of plasma levels, which are 1–2 g/L [18]. We added AAT (0.5 mg/mL) to the ALI culture on the top chamber representing the surface of epithelial cells (apical) or into the culture medium (basolateral)- either alone or together with pro-inflammatory stimuli, such as lipopolysaccharides (LPS) and peptidoglycan (PGN) or LPS/PGN plus freshly isolated human blood neutrophils (Figure 7A). Dependent on the mode of application, AAT either reduced or enhanced CFTR protein levels in ALI cultures exposed to LPS/PGH or LPS/PGN plus neutrophils, and slightly inhibited STAT3 phosphorylation (Figure 7B). Remarkably, ALI cultures treated with AAT alone showed similar levels of CFTR protein than those exposed to AAT in the presence of pro-inflammatory stimuli. As illustrated in Figure 7C, AAT positive cells (in red) were detected in cultures supplemented with exogenous AAT from the apical (inserts 2, 4 and 6) or basolateral sides (inserts 3 and 5), however this was not the case in the culture without added AAT (insert 1). 

**Figure 8:**
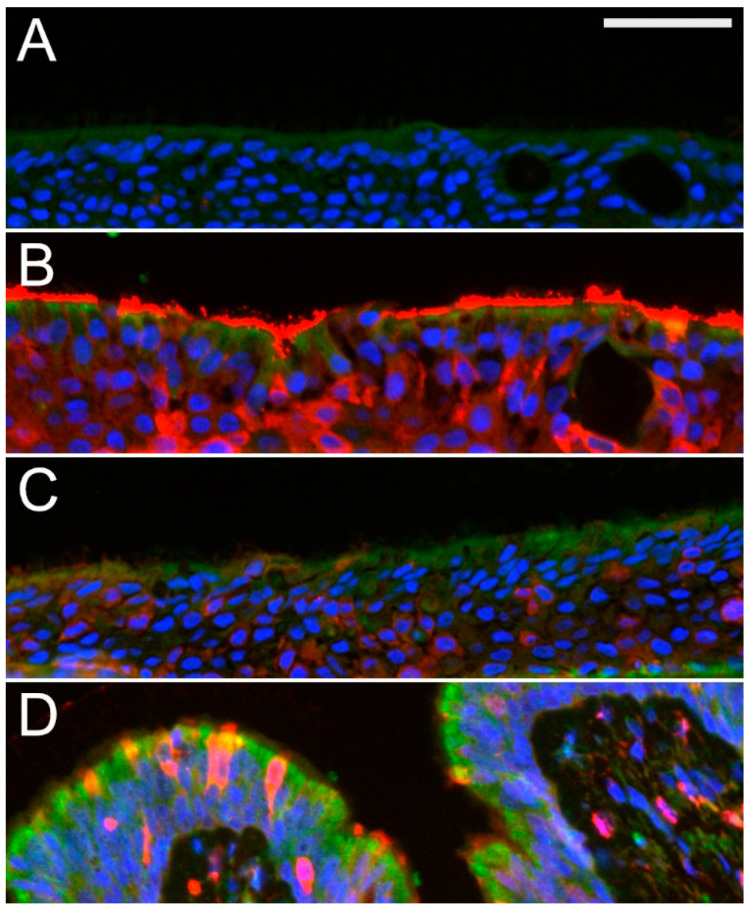
AAT uptake by human bonchial epithelial ALI culture and native human airway epithelium. Combined immunofluorescence for CFTR (green) and AAT (red) highlights typical CFTR positivity in the apical cellular compartment in ALI cultures (**A**–**C**) or bronchial epithelium (**D**). Cell nuclei were visualized with DAPI (blue). In ALI supplemented with 0.5 mg/mL of AAT from the top (**B**) or the bottom (**C**), AAT-positive cells are detected. Similarly, AAT positive cells are detected in intact human bronchial epithelium (**D**). By contrast, no AAT-positive cells are found in cultures without AAT supplementation (**A**). Scale bar is 50 µm.

Further assays revealed that AAT is taken-up by epithelial cells. As shown in Figure 8A, ALI cultures show no positivity for AAT whereas, AAT added apically shows not only a strong staining on the top of the cells but also in the deeper layers (Figure 8B, red). Similarly, basolateral addition of AAT resulted in AAT protein penetration within the ALI cell layers (Figure 8C). These latter results suggest that endogenous AAT can be taken-up by epithelial cells. However, we did not observe significant co-localization between CFTR (in green) and AAT (in red) in ALI epithelium cultures or in human bronchi (Figure 8). It is also worth notifying, that AAT does not induce IL-18 release (data not shown), which is a marker for epithelial damage [19].

## 3. Discussion

Airway epithelium is a physical barrier that covers the respiratory tract and maintains airway tissue integrity and homeostasis [20,21]. CFTR regulates many processes in epithelial physiology, like epithelial surface hydration and luminal pH, among others, and is required for airway epithelial integrity. Various human diseases are associated with defects in epithelium due to the loss of CFTR protein and/or its function [22], and therefore, in vitro models capable of reproducing the key structural and functional aspects of the airway epithelium are powerful tools for studying disease mechanisms and assessment of therapies. 

The cultures of primary human airway epithelial cells grown at air–liquid interface (ALI) are a valuable tool for studying CFTR under normal and pathologic conditions, and for drug testing and/or discovery. Importantly, ALI cultures can be used in co-culture models, for example with innate immune cells. Herein we present ALI cultures based on human explanted lung tracheal epithelial cells that develop a pseudostratified epithelium, consisting of ciliated cells, goblet cells and basal cells, which harbor tight junctions and generate distinct apical and basolateral membranes. One of the major limitations of such ALI model is that the number of available primary cells is low. Indeed, we were able to generate only 3 to 6 ALI inserts from one donor. To overcome this problem, we developed an approach allowing obtaining material for immunocytochemistry, protein and RNA analysis from every single ALI insert (Figure 2). The data obtained from the different assays applied to the same set of ALI inserts would allow personalized studies on inflammation, immunology and/or drug delivery.

In this study, we specifically aimed to show the presence of CFTR protein in ALI cultures and to test if AAT protein, which is used as biopharmaceutical, has any effects on CFTR levels. Indeed, histological examination of formalin- or Methacarn-fixed, paraffin-embedded ALI sections revealed that our cell cultures display a phenotypic resemblance to the lower airway epithelium. They formed pseudostratified epithelium and exhibited CFTR protein expression at the apical surface of ciliated cells (Figure 3 and Figure 4). It is worth noting, that the ALI cultures, similarly like in native epithelia from human lung tissue, contain CFTR rich cells compatible with ionocytes described in human and murine lung tissues [23,24]. By using single-cell sequencing technology, and mapping cell types of the tissue, researchers recently discovered this rare cell type that was named “pulmonary ionocyte” because of the gene expression pattern similarity to ionocytes (cells that regulate ion transport and hydration in fish gills and frog skin) [23,24]. Human ionocytes comprise about 0.5–1.5% of epithelial cells, specifically express FOXI1, achaete-scute family bHLH transcription factor (ASCL3) and CFTR and are involved in the fluid regulation at the epithelial interface [23,24]. For decades, researchers have assumed that only ciliated cells express CFTR whereas according to the new data, the majority of CFTR expression seems to occur in ionocytes [23,24]. This underscores how important ionocytes are for airway-surface regulation and encourages further studies regarding ionocyte expression in ALI cultures derived from different human lung donors. 

To investigate whether AAT has any effect on CFTR levels, we first employed human colon carcinoma T84 cell line. Indeed, T84 cells exposed to constant amount of human AAT (0.5 mg/mL) markedly upregulated CFTR protein levels. On the other hand, transcriptome analysis revealed 226 genes (45 down- and 181 up regulated) differently regulated in T84 cells treated with AAT versus non-treated controls but no effect on *CFTR* expression was found (data not shown). Among the up-regulated genes were MYH13, KRT80 and TUBB3, all of which proposed as interacting partners of CFTR [7]. 

The finding that AAT regulates CFTR protein levels in T84 cells, we replicated in human ALI inserts generated from lung donor. Notably, effect of AAT on CFTR upregulation was independent on the presence of pro-inflammatory stimuli (like LPS/PGN) alone or with neutrophils. Even though CFTR interacts with different proteins [25], we did not find any co-localization of AAT with CFTR suggesting that any functional coupling is independent of their direct association. Current data support a specific contribution of different kinases in the regulation of CFTR levels and function [25,26]. In line, we found that in parallel to the regulation of CFTR, AAT inhibits STAT3 activation, which is one of the CF modifier genes [5,27]. This latter finding highlights a putative relationship between CFTR, AAT and STAT3 signaling pathway. 

To our knowledge, we provide a first evidence that AAT may positively influence the evolution of airways diseases via regulation of CFTR protein levels. Currently we are not able to rule out if effects of AAT concentration and time-dependent and if biological activities of AAT differ when applied into different cellular compartments. Lockett and co-authors reported that polarized primary human lung epithelial cells take-up basolaterally supplied AAT that is secreted apically [28]. Likewise, we show that ALI cultures basolaterally supplemented with AAT possess a similar AAT-positivity as human native bronchial epithelium cells whereas much stronger AAT-positivity can be detected when AAT applied apically (Figure 8). In order to answer what concentration and route for delivering AAT is worthwhile to explore in the future, and whether AAT affects functional activity of CFTR, electrophysiological and surface protein-specific labeling assays need to be performed. 

In general, beneficial effects of AAT therapy have been demonstrated not only in patients with AAT deficiency-related lung emphysema, but also in patients with CF [9,14]. Moreover, the therapy with AAT seems to be promising in other human diseases, including panniculitis, arthritis, stroke, sepsis, graft-versus host disease and covid-19 [9,29,30]. For instance, AAT was reported to inhibit a wide range of proteinases, and to interact with inflammatory mediators (e.g., IL-8, TNF-α receptors, free heme and leukotriene B4) and block their actions at the inflammation sites [31]. Experimental and clinical studies in CF suggest that an impaired bacteria killing and the accumulation of neutrophils enhance inflammatory reaction [3]. Hence, it is possible that the above-mentioned biological activities of AAT, together with our discovered its effect on CFTR levels, might be responsible for the beneficial effects in CF and other pathological conditions. At this point, human lung epithelial ALI cultures alone or in co-culture with neutrophils provide valuable tool for CFTR analysis.

The epithelial ALI system is an established in vitro model that is used to study the differentiation and function of airway epithelial cells [32]. Due to the easier availability, the rodent lung bronchial epithelia cells have been predominantly used as a source of primary cells. However, species-specific differences pose concerns when studying complex mechanisms of human respiratory diseases. Significant differences in the anatomy and physiology between rodents and humans, and an in-equivalence between human diseases and animal models of human respiratory diseases, such as asthma, chronic obstructive pulmonary disease and others, lead to high failure rates of clinical trials designed using animal data [33]. In recent years, it is increasingly recognized the limited success in translating animal data to human. To this end, others and we prefer to study CFTR in differentiated ALI cultures of human airway epithelial cells, which contain all major cell types present in human airway epithelium [32,34]. Human epithelial ALI models have barrier properties and metabolic functions similar to those found in vivo and allow studying the variability in donor-based responses. Epithelial cultures generated from different donors may help to reveal the impact of the clinical history of the individual patient. On the other hand, factors that are shared across ALI cultures from different patients may help to defined useful therapeutic targets. Here we proposed a protocol allowing parallel performance of various analyses from the single ALI insert. We believe that this approach can improve possibilities to combine ALI characteristics with patient’s clinical features. 

## 4. Materials and Methods

### 4.1. Bronchial Epithelial Cells at Air-Liquid Interface (ALI) Culture

Primary human bronchial epithelial cells (PBEC) were isolated from fresh tissues that were obtained during tumor resections or lung transplantation with full consent of the patients (Ethics approval: ethics committee Hannover Medical School, no. 2699–2015). Tissue material from donor trachea or explanted lung was stored up to 16 h in RPMI supplemented with Penicillin/Strepavidin and Amphotericin B at 4 °C.

The method for the generation of PBEC ALI cultures was adopted from the well-established ALI culture protocols from The University of North Carolina at Chapel Hill [16] and from The University of Leiden, The Netherlands [15]. Briefly, tracheas were opened and pachenchymal tissue was removed from bronchi [32]. Airways were subjected to protease type XIV (Merck, Darmstadt, Germany) digest (0.18% in HBSS without Ca^2+^ and without Mg^2+^) for 2 h at 37 °C [15]. The epithelial cell layer was scraped from the surface of trachea or bronchus with the backside of a scalpel. Following the Leiden University protocol the cells were expanded in modified keratinocyte medium (Gibco, Thermo Fisher Scientific, Waltham, MA, USA) [15]. Sometimes cell isolation and expansion was perfomed according to the Chapel Hill protocol [16]. Here, epithelial cells were applied to an additional DNase I (Sigma-Aldrich, St. Louis, MO, USA) digestion step before seeding and expanded in Bronchial Epithelial Cell Growth Basal Medium (BEGM, StemCell Technologies, Vancouver, BC, Canada) supplemented with Nu-serum growth medium supplement (Corning, Corning, NY, USA). Nu-serum containing BEGM was replaced gradually by retinoic acid containing BEGM [16].

Independent of the cell isolation protocol, for cells derived from tracheas the medium was supplemented with Fungizone (Amphotericin B, Thermofisher Scientific) during the first four days of expansion culture to prevent contaminations with fungi. When confluency was reached adherent cells were detached using 0.25% trypsin-EDTA solution (Gibco, Thermo Fisher Scientific) for 5 min at 37 °C following and subsequent addition of 1 mg/mL soybean trypsin inhibitor (Sigma-Aldrich) in HBSS. Remaining strongly adherent cells were subjected to a second trypsinization step (2 min, 37 °C) as suggested by Fulcher et al. [32]. Detached cells were collected by centrifugation and seeded in a cell density of 2.5 to 3.5 × 10^5^ cells per 200 µL of BEGM for ALI (composition decribed in detail by [16]) per insert of a 12-well ALI plate (0.4 µm pore polyethylene terephtalate membrane inserts, Corning) with 720 µL BEGM ALI medium provided basolaterally. When the cell layer appeared confluent the apical medium was completely removed. Epithelial cell layers were allowed to differentiate at ALI with Pneumacult ALI medium (Stemcell technologies) for at least three weeks until cilia beating could be observed under the microscope.

### 4.2. Histological Analysis

ALI cultures were fixed in 4% buffered formaldehyde for 24 h or with methanol-Carnoy’s solution (Methacarn, 60% methanol, 30% chloroform, 10% glacial acetic acid) for 48 h to preserve mucus [35]. After paraffin embedding following routine histologic protocols, 2 µm thick sections were cut. These sections were deparaffinized in xylene and rehydrated following standard procedures. From formaldehyde fixed samples hematoxylin and eosin (H&E) stainings were prepared for morphologic evaluation. For periodic acid-Schiff (PAS) staining, sections of methacarn-fixed ALI cultures were deparaffinized, treated with 1% (*w/v*) periodic acid (Merck, Darmstadt, Germany), then stained with Schiff’s reagent (Sigma-Aldrich Sigma-Aldrich, St. Louis, MO, USA) and counterstained with hematoxylin (Merck).

### 4.3. Immunofluorescence

Immunofluorescence stainings were performed after heat induced antigen retrieval at pH 9.0 (HIER Tris-EDTA Buffer, Zytomed Systems, Bargteheide, Germany) and blocking with normal donkey serum (Jackson ImmunoResearch, Ely, UK). Primary antibodies (see Table 1) diluted in the kit supplied dilution buffer (Antibody Diluent, Zytomed Systems) were applied for 30 min and detected with specific secondary antibodies. Stained slides were mounted with DAPI supplemented mounting medium (DAPI/DuraTect-Solution, ZytoVision, Bremerhaven, Germany) and imaged on an automated fluorescence microscope with a 40× objective (BZ-9000, Keyence, Osaka, Japan). For the peptide competition assay, the primary antibody was pre-incubated with the CFTR-specific peptide against which it was raised (NBD2 1204-1211 of CFTR, H-WPSGGQMT-OH; Kaneka Eurogentec S.A., Seraing, Belgium) at a final concentration of 1 µg/µL in antibody dilution buffer (Zytomed Systems) for 30 min.

### 4.4. T84 Cell Culture

The intestinal cancer epithelial cell line T84, highly expressing CFTR, and commonly used in the experiments to study the modulation of CFTR, was grown in Dulbecco’s Modified Eagle Medium (DMEM/F12, Thermo Fisher Scientific, Waltham, MA, USA) supplemented with 10% fetal calf serum (FCS) and 1% of 100× penicillin-streptavidin-glutamine-solution (Thermo Fisher Scientific). Presented data are from confluent T84 cell layers grown submerged in 35 mm diameter petri dishes.

### 4.5. Western Blot

SDS-rich whole cell lysates were prepared using Laemmli buffer (2% SDS, 10% glycerol) to homogenize cell layers of ALI cultures. To detect CFTR, lysates were incubated for 30 min at 37 °C prior to loading on a denaturing 6% polyacrylamide gel at 4 °C for 18 h until the 72 kDa size marker band approached end of the acrylamide matrix. Transfer to a nitrocellulose (Hybond C, GE Healthcare, Chicago, IL, USA) membrane was done immersed in an ice bath at 0 °C for 18 h. StartingBlock Block Buffer (Thermo Fisher Scientific, Waltham, MA, USA) was used for blocking for 2 h at room temperature. CFTR detection was done using four CFTR monoclonal antibodies provided by T. Jensen, Chapel Hill via the antibody distribution program hosted by the CF foundation (see Table 2) in a mixture of 1:1:1:1. Depending on signal intensity, two different HRP substrate solutions (SuperSignal West Pico or SuperSignal West Femto, Thermo Fisher Scientific) were applied onto membranes. Fatty acid synthase (FASN, MW 273 kDa) was used as a loading control. Moreover, to compare non-treated controls and AAT-treated T84 cells we used paired samples from the same experiment.

### 4.6. Statistical Analyses

Normally distributed data were presented as mean (SD). For the comparison of more than two groups one way ANOVA was used. For two related samples which fail the normality test the nonparametric Wilcoxon signed rank test was performed. A *p*-value of less than 0.05 was considered significant. Statistics and visualisation of the data was performed with GraphPad Prism 5 (GraphPad Software, San Diego, CA, USA).

## 5. Conclusions

In this study, we present successfully established protocol in providing the biomaterial for protein, RNA and histology analysis from each human epithelial ALI insert. By employing our ALI model, we for the first time were able to demonstrate that AAT can alter CFTR protein levels. While AAT augmentation therapies (intravenous or aerosolized form) proved to be safe and well tolerated in emphysema patients with inherited AAT deficiency, the therapeutic benefit in CF lung disease remains to be determined. *SERPINA1* gene, encoding AAT protein, is known as a modifying gene of cystic fibrosis whereby our data allow speculating that AAT may facilitate CFTR-mediated epithelial fluid secretion and improve mucociliary clearance. The therapy with AAT, as mimicked by apical and basolateral application in ALI cultures, might be beneficial for CFTR processing and maturation. Currently, however, we are not able to answer if AAT directly regulates the synthesis of CFTR or stabilizes the CFTR protein. Further studies, including single cell CFTR expression in response to AAT, will answer these questions.

## Figures and Tables

**Table 1:** Antibodies used for immunofluorescence experiments.

Antibody	Host/Clonality	Dilution	Company	Cat. No.
AcTubulin	Mouse/ Monoclonal	1:5000	Sigma-Aldrich (St. Louis, MO, USA)	T6793
Muc5AC	Rabbit/ Polyclonal	1:500	Abcam (Cambridge, UK)	ab78660
Alexa Fluor 488	Donkey- anti-rabbit	1:200	Abcam (Cambridge, UK)	ab150061
Alexa Fluor 594	Donkey- anti-mouse	1:200	Thermo Fisher Scientific (Waltham, MA, USA)	R37115
CFTR	Mouse/ Monoclonal	1:200	CF Foundation (Bethesda, MD, USA)	596
ZO1	Goat/ Polyclonal	1:200	Abcam (Cambridge, UK)	ab99462
FOXI1	Rabbit/ Polyclonal	1:100	Atlas Antibodies, (Bromma, Sweden)	HPA071469
Cyanine 3	Donkey- anti-mouse	1:200	Dianova (Hamburg, Germany)	715-165-151
Alexa Fluor 488	Donkey- anti-goat	1:200	Abcam (Cambridge, UK)	ab150105
Alexa Fluor 555	Donkey- anti-mouse	1:200	Abcam (Cambridge, UK)	ab150106

**Table 2:** Antibodies used for Western Blot analysis.

Antibody	Host/Clonality	Dilution	Company	Cat. No.
CFTR	Mouse/ Monoclonal	1:400	CF Foundation (Bethesda, MD, USA)	217, 660, 570, 596
AAT	Rabbit/ Polyclonal	1:800	Agilent Dako (Santa Clara, CA, USA)	A0012
Phospho- STAT3Y705	Rabbit/ Monoclonal	1:2000	Abcam (Cambridge, UK)	ab76315
FASN (HRP)	Rabbit/ Monoclonal	1:5000	Abcam (Cambridge, UK)	ab196854
IgG H&L (HRP)	Goat- anti-Mouse	1:5000	Abcam (Cambridge, UK)	ab97040
IgG H&L (HRP)	Goat- anti-Rabbit	1:2000	Abcam (Cambridge, UK)	ab205718

## Data Availability

The datasets used and analyzed during the current study are available from the corresponding author on reasonable request.
